# Preparation of Monoclonal Antibody and Development of Indirect Competitive Enzyme-Linked Immunosorbent Assay and Fluorescence-Linked Immunosorbent Assay for Detecting 3-Amino-5-methylmorpholino-2-oxazolidinone (AMOZ) in Edible Animal Tissue

**DOI:** 10.3390/molecules26144243

**Published:** 2021-07-13

**Authors:** Yong Xie, Yarong Wang, Xueling Yan, Lu Gan, Tao Le

**Affiliations:** 1 Bioassay 3D Reconstruction Laboratory, Chongqing College of Electronic Engineering, Chongqing 401331, China; yongxie88@163.com (Y.X.); ganlu6767@163.com (L.G.); 2 College of Life Sciences, Chongqing Normal University, Chongqing 401331, China; wang6521475211@163.com (Y.W.); littleoutman@126.com (X.Y.)

**Keywords:** 3-amino-5-methylmorpholino-2-oxazolidinone, monoclonal antibody, ic-ELISA, FLISA, animal tissue

## Abstract

To monitor the illegal used of furaltadone, a highly sensitive indirect competitive enzyme-linked immunosorbent assay (ic-ELISA) and fluorescence-linked immunosorbent assay (FLISA) based on a monoclonal antibody (mAb) were developed for the detection of 3-amino-5-methylmorpholino-2-oxazolidinone (AMOZ), the major metabolite of furaltadone in animal tissues. The highly specific mAb, which was very sensitive to a nitrophenyl derivative of AMOZ (2-NP-AMOZ) with IC_50_ values of 0.11 and 0.09 ng/mL for ic-ELISA and FLISA, respectively, was selected for the development of immunoassays. For both the ic-ELISA and FLISA for AMOZ-spiked experiments, acceptable recovery rates of 81.1–105.3% and coefficients of variation of 4.7–9.8% were obtained. In addition, results from both ic-ELISA and FLISA methods for spiked samples’ data showed excellent correlation coefficients ranging from 0.9652 to 0.9927. Meanwhile, the proposed ic-ELISA and FLISA for thirty spiked samples were confirmed by standard LC-MS/MS with high correlation coefficients of 0.9911 and 0.9921, respectively. These results suggest that the developed ic-ELISA and FLISA are valid and cost-effective tools for high-throughput monitoring methods for AMOZ residues in animal tissues.

## 1. Introduction

**Figure 1:**
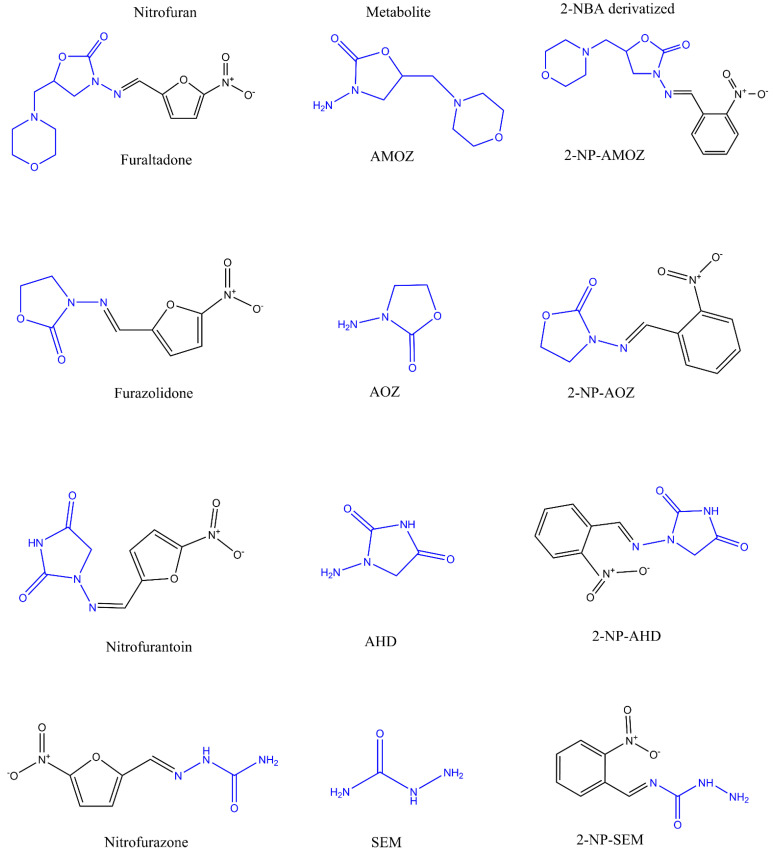
Column-by-column, from left to right, the molecular structures of the parent nitrofuran drugs, nitrofuran metabolites after in vivo transformation, and nitrofuran metabolites after derivatization with 2-nitrobenzaldehyde.

**Figure 2:**
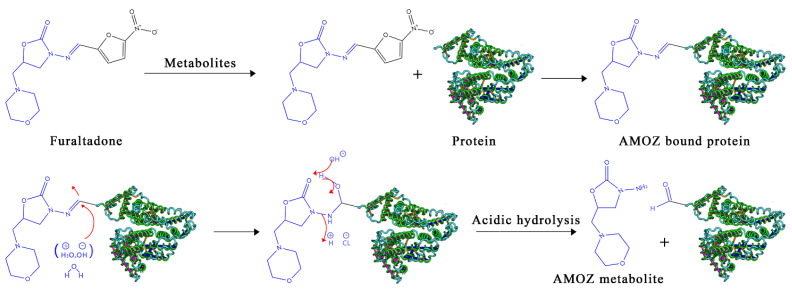
Description of the conversion of the parent nitrofuran to its metabolites in vivo, the formation of metabolite–protein adducts, and the acid hydrolysis of metabolite–protein adducts to release metabolites.

Nitrofuran antibiotics, such as furaltadone, furazolidone, nitrofurantoin, and nitrofurazone (Figure 1), are widely used in gastrointestinal and dermatological microbiological infections that often occur in farmed bees, swine, cattle, poultry, fish, and shrimp [1]. However, this family of drugs and their metabolites are known to be carcinogenic and mutagenic [2,3], and as a result, the nitrofurans have been listed as prohibited veterinary drugs in farmed animals for food production by many countries and regions, including the European Union (EU), USA, and China [4]. Meanwhile, many studies have demonstrated that the nitrofurans are quite unstable in vivo and are rapidly metabolized [5,6,7]. The metabolites formed from the above nitrofurans are 3-amino-5-morpholinomethyl-2-oxazolidinone (AMOZ), 3-amino-2-oxazolidinone (AOZ), 1-aminohydantoin (AHD), and semicarbazide (SEM) (Figure 1). Over 12 h after administration, the residues of parent compounds in edible tissues drop below detectable levels, but these toxic metabolites then bind to tissue protein, forming metabolite–protein adducts that are stable for considerable periods in animal tissues (Figure 2) [8,9]. These metabolites of nitrofurans have been recognized as a marker residue in edible tissues for evaluation of nitrofurans [10,11].

**Figure 3:**
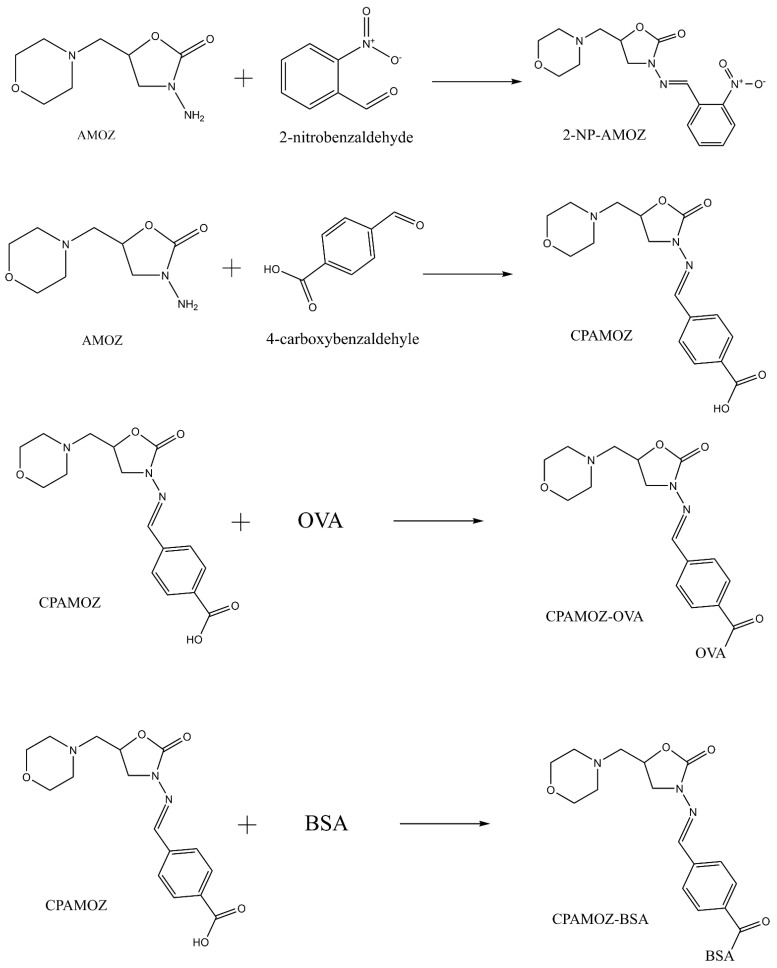
Routes of synthesis of CPAMOZ, 2-NP-AMOZ, CPAMOZ-BSA, and CPAMOZ-OVA. CPAMOZ and 2-NP-AMOZ were, respectively, derived with 4-carboxybenzaldehyle and 2-nitrobenzaldehyde from AMOZ. CPAMOZ was used to synthesize the immunogen and coating antigen, while 2-NP-AMOZ was the target sample derivative.

Therefore, monitoring residues of furaltadone in edible tissues is unsuitable because of its instability in vivo. Analytical methods for detecting AMOZ have been developed instead of detecting furaltadone in food matrices. As AMOZ covalently binds to proteins, the analysis usually entails releasing the metabolite from the tissues under acidic conditions (Figure 2) and a derivatization step with 2-nitrobenzaldehyde to form nitrophenyl derivative (2-NP-AMOZ) before detection (Figure 3). 

A variety of instrumental methods for the determination of furaltadone and AMOZ residues in various animal-derived foods have been reported in the past decades. Due to the accuracy and precision, liquid chromatography with ultraviolet [12], liquid chromatography–mass spectrometry [1,13,14,15,16], and liquid chromatography–tandem mass spectrometry (LC-MS/MS) [1,17] have been widely used. However, these methods generally require complex sample pretreatment procedures, special equipment, trained professionals, and extensive clean-up procedures.

In comparison, immunological methods offer fast, accurate, specific, and inexpensive detection solutions. For example, many immunological methods have been used to detect AMOZ, such as the immunochromatographic assay [18,19], indirect competitive enzyme-linked immunosorbent assay (ic-ELISA) [3,7,20,21,22], chemiluminescent ELISA [8], fluorescence polarization immunoassay [23], and time-resolved fluoroimmunoassay [24]. Among them, the ELISA is one of the most popular methods for the simple high-throughput screening of AMOZ residue in food samples. However, traditional colorimetric detection ELISAs suffer from low sensitivity, and they require additional sample pretreatment to enrich and purify AMOZ in complex samples. Numerous strategies, including the immunochromatographic assay, fluorescence polarization immunoassay, and time-resolved fluoroimmunoassay, have the characteristics of simple sample preparation, short analysis time, and high detection sensitivity. However, conventional fluorescent probes are susceptible to interference from nonspecific fluorescence, particularly when measured on biological samples with high background fluorescence [25,26].

Quantum dots (QDs), a family of fluorescent semiconductor nanocrystals, are considered promising reporters for immunoassays due to their unique optical properties, including high quantum yield, high photostability, large molar extinction coefficient, broad absorption, and narrow fluorescent emission spectrum [2,25]. Recently, QDs have been used in biomolecule detections [27,28,29] and have been established as an attractive option as fluorescent probes for immunoassays [2,30,31,32]. However, the application of a QD-based fluorescence-linked immunosorbent assay (FLISA) for the detection of AMOZ has not yet been reported.

In this study, novel sensitive and specific monoclonal antibodies (mAbs) against 2-NP-AMOZ were prepared with modification using the semi-solid medium screening of four monoclonal antibody cell lines. With the mAbs, indirect competitive ELISA (ic-ELISA) and QD-based FLISA were optimized and established, which were employed as a reliable, sensitive, and rapid detection method for monitoring AMOZ residues at trace levels in various tissue samples. The proposed ic-ELISA and FLISA were validated by conventional LC-MS/MS with good correlation.

## 2. Materials and Methods

### 2.1. Materials and Reagents

Furaltadone, furazolidone, nitrofurantoin, furaltadone, AMOZ, AOZ, AHD, SEM, 2-NP-AMOZ, 2-NP-AOZ, 2-NP-AHD, 2-NP-SEM, 2-nitrobenzaldehyde, 4-carboxybenzaldehyle, and horseradish peroxidase-labeled goat anti-mouse IgG were purchased from Sigma-Aldrich (St. Louis, MO, USA). Goat anti-mouse IgG conjugated with 655 nm QDs was obtained from Invitrogen Corp (Carlsbad, CA, USA). *N,N*-dicyclohexylcarbodiimide (DCC), *N,N*-dimethylformamide (DMF), *N*-hydroxysuccinimide (NHS), dimethyl sulfoxide (DMSO), ethyl acetate, and all other chemicals were of analytical grade and purchased from Sinopharm Chemical Reagent Co. Ltd. (Beijing, China) unless indicated otherwise.

### 2.2. Preparation of AMOZ-Protein Conjugate

To prepare the AMOZ monoclonal antibody, AMOZ was initially used to prepare carboxyphenyl AMOZ (CPAMOZ), which was further derivatized to conjugate bovine serum albumin (BSA) and ovalbumin (OVA) to prepare the immunogen and coating antigen, respectively, as shown in Figure 3. To prepare CPAMOZ, 1.5 mmol of 4-carboxybenzaldehyle was dissolved in 5 mL of dried methanol, to which 1.0 mmol of AMOZ was subsequently injected. The reaction mixture was refluxed at 65 °C overnight, and the formation of CPAMOZ was confirmed by thin-layer chromatography. The resulting CPAMOZ was precipitated and washed with ethanol and finally dried in vacuum at room temperature. Furthermore, to prepare CPAMOZ-BSA and CPAMOZ-OVA conjugates, CPAMOZ (10 μmol), NHS (20 μmol), and DCC (20 μmol) were reacted in 500 µL of DMF for 12 h at room temperature. Subsequently, the activated CPAMOZ was centrifuged at 2500 g for 10 min, and the supernatant was added dropwise under stirring to 90 mg of BSA (or 60 mg of OVA) in 9.5 mL of PBS (pH 7.4). The conjugation mixture was stirred at 4 °C overnight and dialyzed against three changes of 0.1 M of PBS (pH 7.4) at 4 °C for 72 h under constant agitation. Finally, the purified CPAMOZ-protein conjugations (CPAMOZ-BSA or CPAMOZ-OVA) were stored at −20 °C for future use.

### 2.3. Production of mAb by Hybridoma Approach

All animal experiments in this study adhered to the Chongqing Normal University animal experiment center guidelines and were approved by the Animal Ethics Committee (CQNU-2015-02-003). Eight BALB/c female mice (6–8 weeks old, Chongqing Center for Disease Control and Prevention) were immunized with 100 μg of CPAMOZ-BSA conjugates in Freund’s complete adjuvant. Three weeks later, two subsequent boost injections were given at 2 week intervals with the same dosage of immunogen, emulsified in Freund’s incomplete adjuvant. Two weeks after the last immunization, the mice were tail-bled, and the titer and specificity of the antiserum were determined by a noncompetitive indirect ELISA. The mouse with the highest anti-2-NP-AMOZ titer was selected for cell fusion. Cell fusion procedures were carried out as we previously reported [9]. Specifically, hybridoma cell lines were obtained by fusing Sp2/00 myeloma cells with spleen cells from the selected mouse at a ratio of 5–10:1. The fused cells were first treated with a selective liquid medium, the culture supernatants from the wells with hybridomas were assayed, and then the positive wells were subcloned in the following subcloning procedure with the semisolid medium sequentially used [26]. Cells in those positive wells were each suspended in the semisolid medium and distributed into 6-well plates. Subsequently, an ic-ELISA was used to screen for positive hybridomas. After the cell culture, the selected hybridoma cells were injected intraperitoneally into mice to generate mAbs in ascites fluid. The mAb were purified from ascitic fluid by ammonium sulphate precipitation using a G protein affinity column (Amersham Biosciences, Uppsala, Sweden). The purified mAb was stored at −20 °C until future use.

### 2.4. Procedure of ic-ELISA

To carry out 2-NP-AMOZ ic-ELISA, 96-well plates were first coated with 100 μL of CPAMOZ-OVA in 50 mM of carbonate buffer solution (pH = 9.6) overnight at 4 °C. The plates were then washed three times with PBS containing 0.05% Tween 20 (PBST, pH = 7.4) and subsequently blocked with 1% OVA in PBS at 37 °C for 1 h. The plates were washed again three times, after which 50 μL of different concentrations of 2-NP-AMOZ analytes and 50 μL of the obtained mAb were added into each well, incubated at 37 °C for 1 h, and the plates were washed three times with the PBST. Subsequently, 100 μL of horseradish peroxidase-labeled goat anti-mouse IgG (1:10,000 dilution) was added to the wells and incubated at 37 °C for 1 h. This was followed by a washing step of three washes with PBST solution, after which 100 μL of substrate solution was added to each well and incubated for 15 min in darkness. The reaction was stopped by adding 50 μL/well of 2 M of H_2_SO_4._ The absorbance at 450 nm was measured on a Tecan Sunrise 2.5 microplate reader (SUNRISE, Austria). A calibration curve was constructed by plotting (B/B_0_) × 100% vs. log C, where B and B_0_ represent the absorbance of the analyte at the known concentrates (standards) and at zero concentration of the analyte, respectively.

### 2.5. Procedure of FLISA

The procedures for FLISA and ic-ELISA were identical before the secondary antibody was added. More specifically, after washing with PBST for three times, 100 μL of 655 nm QDs-conjugated goat anti-mouse IgG (1:10,000 dilution) was added to each well and incubated at 37 °C for 45 min. After three washes with PBST, a SpectraMax M2e Microplate Reader (Molecular Devices, San Jose, CA, USA) was used to measure the fluorescence signals from samples with excitation/emission at 360/655 nm. The fluorescence intensities were identified as B_0_ and B for the control and testing sample. The standard curve was established by plotting the form (B/B_0_) 100% against the 2-NP-AMOZ concentration.

### 2.6. Determination of Cross-Reactivity

The extent of cross-reactivity was evaluated by determining IC_50_ values using an ic-ELISA as described above. Several nitrofuran analogs, including nitrofurantoin, furazolidone, furaltadone, nitrofurazone, and their metabolites, including AHD, AOZ, SEM, 2-NP-AMOZ, 2-NP-AHD, 2-NP-AOZ, and 2-NP-SEM, were selected for cross-reactivity testing. The concentration of the standard solutions of the compounds was set from 0.01 to 1000 ng/mL. The cross-reactivity values were calculated as follows: % cross-reactivity = [(IC_50_ value of 2-NP-AMOZ)/(IC_50_ value of compound being tested)] × 100%.

### 2.7. Samples Preparation

Edible animal tissue samples including muscle and liver tissues from catfish, sturgeon, shrimp, carp, chicken, and swine were obtained from a local supermarket. One gram of each edible tissue was separately homogenized, and the homogenates were collected inside of a 50 mL centrifuge tube. The samples were spiked with AMOZ at concentrations of 0.5, 1.0, and 5.0 μg/kg, based on the AMOZ residue level limits set by the European Union [33]. To each of the spiked samples, 20 mL of ethyl acetate was added, and the resulting mixture was vortexed and subsequently treated in a boiling water bath for 10 min. The samples were extracted by mixing with 4 mL of deionized water, 0.5 mL of 1 M of HCl, and 100 μL of 50 mM of 2-nitrobenzaldehyde in DMSO. Furthermore, the extracted solution was centrifuged at 5000 rpm for 20 min at 4 °C, and the ethyl acetate fraction was collected and dried under a stream of nitrogen at 50–60 °C. Finally, the residue was reconstituted in 2 mL of hexane and 1 mL of PBS (0.1 M, pH 7.4), centrifuged at 4000 rpm for 5 min, reconstituted in 1 mL of PBS, and analyzed by both ic-ELISA and FLISA.

### 2.8. Validation of the ic-ELISA and FLISA Method

Both ic-ELISA and FLISA detections were performed using AMOZ-spiked samples in different food matrices, including in catfish, sturgeon, shrimp, crab, and the muscle and liver of swine and chicken. All food matrices were initially tested by LC-MS/MS [14] to be free of AMOZ compounds before being spiked with AMOZ. To calculate the recovery of the spiked AMOZ from different tissue matrices, blank tissue samples were spiked with AMOZ at levels of 0.5, 1.0, and 5.0 μg/kg (n = 5 at each concentration), and the recovery was calculated by the following equation: (measured concentration/spiked concentration) × 100%. The coefficient of variation (CV) was determined from the analysis of the above samples spiked with AMOZ. The analysis of each concentration level was repeated in triplicate over a time span of two months. Furthermore, 20 blank samples were employed for LOD determination.

To evaluate the reliability of the developed ic-ELISA and FLISA for incurred samples, comparisons of ic-ELISA and FLISA with LC-MS/MS were carried out using the samples from an animal feeding experiment. The individual eggs were homogenized and stored at −20 °C until they were subjected to ic-ELISA, FLISA, and LC-MS/MS analyses. The LC-MS/MS procedure is described in the literature [14].

## 3. Results and Discussion

### 3.1. Design and Synthesis of Haptens

The rational design of haptens is a critical factor to the immunoassays and the successful preparation of highly specific antibodies against small-molecular-weight analytes. As the furaltadone antibiotic is highly unstable in vivo, and its metabolite can bind to tissue proteins to form stable compounds with a long residence time, the detection of illegal uses of furaltadone in farmed animal feed generally involves the detection of furaltadone metabolite concentrations in animal tissues. In this study, AMOZ, the stable metabolite of furaltadone, was used as the desired hapten was to generate monoclonal antibodies. However, AMOZ has only one amino group available for coupling the hapten to a protein carrier, which were designed by derivatizing furaltadone metabolites with 3-carboxybenzaldehyde or 2-(3-formylphenoxy)acetic acid to generate immunogens [7,19,20,34]. In the present study, AMOZ was reacted directly with 4-carboxybenzaldehyle, instead of 3-carboxybenzaldehyle and 2-(3-formylphenoxy) acetic acid, to form novel hapten CPAMOZ. Compared with other immunogens reported in the literature, this method produces monoclonal antibodies with higher specificity and sensitivity. The generation of CPAMOZ with the carboxylic group was confirmed by NMR spectroscopy. Then, CPAMOZ was coupled to carrier proteins BSA and OVA for the immunogen and coating antigen, respectively. The routes of synthesis are shown in Figure 3. CPAMOZ, BSA, OVA, CPAMOZ-OVA, and CPAMOZ-BSA were determined using UV-VIS spectroscopy. The results showed their wavelengths of maximum absorbance to be 294, 280, 278, 291, and 291 nm, respectively, suggesting that CPAMOZ had been successfully coupled with the carrier protein. The optimal molar ratio of hapten to carrier proteins for CPAMOZ-BSA and CPAMOZ-OVA was 20:1 and 5:1 for BSA and OVA, respectively.

### 3.2. Screening of Monoclonal Antibodies

In this study, the AMOZ was derivatized to form CPAMOZ, which offered a carboxyl group to form an amide bond with the amino group of carrier proteins and a structure similar to 2-NP-AMOZ, the target molecule of the detection. This linker was advantageous to expose the molecular structure and stimulate the animals to create specific antibodies. We have developed different molar ratios of CPAMOZ to BSA to determine the effect of various hapten-to-protein molar ratios on antibody sensitivity. The obtained results showed that BSA is a suitable carrier protein for CPAMOZ, and a molar ratio of CPAMOZ to BSA of about 20:1 produced a good immunogen that induced a significant immune response in mice. After cell fusion and culture, hybridomas secreting specific mAbs directed against 2-NP-AMOZ were identified by ic-ELISA using CPAMOZ-OVA as a coating antigen. Several clones were obtained, and they were screened for the most sensitive antibody based on both the IC_50_ and LOD values. After several cell fusions and cultures, a total of 20 positive clones were screened out by the screening procedure. At last, four clones (5C10/3A8, 6D8, 4E11/B3, and 5A7/8E) that had an intense reaction with 2-NP-AMOZ were subcloned three times using semisolid medium. After subcloning, a stable hybridoma, clone 5C10/3A8, exhibited a higher sensitivity and specificity than the other mAbs. The mAb 5C10/3A8 was determined to be the IgG2a isotype and featured kappa (K) light chains. Thus, it was finally chosen for mAb production in mice ascites and for the establishment of ic-ELISA and FLISA.

### 3.3. Standard Curve and Cross-Reactivity for the ic-ELISA and FLISA

**Figure 4:**
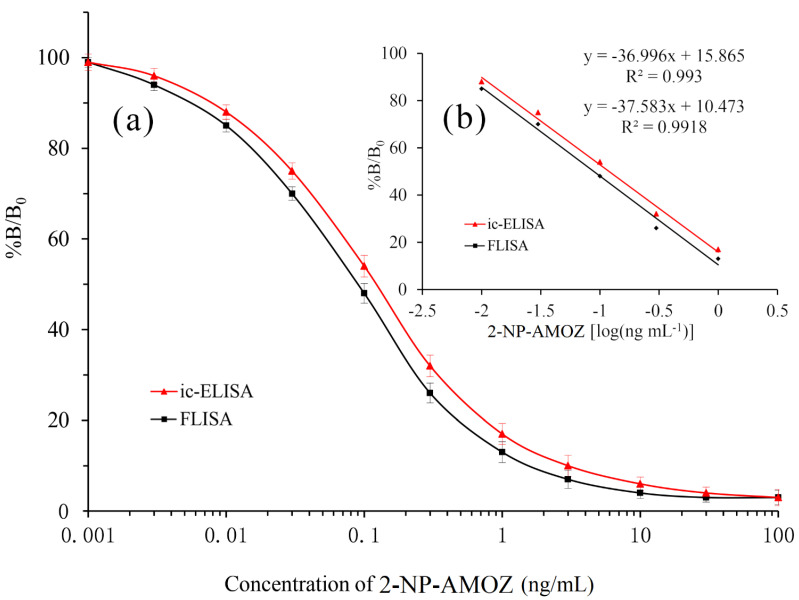
Standard curves of ic-ELISA and FLISA for 2-NP-AMOZ detection (n = 5). (**a**) A typical calibration curve illustrated by plotting (B/B_0_) against 2-NP-AMOZ concentration. (**b**) The linear portion of the standard curve. B and B_0_ are the absorbances of the sample with/without 2-NP-AMOZ, respectively.

The AMOZ residue was derivatized by the proposed method to form 2-NP-AMOZ for detection. Furthermore, the inhibition standard curve of 2-NP-AMOZ was obtained. As demonstrated in Figure 4a, representative ic-ELISA and FLISA standard curves were constructed over a 2-NP-AMOZ concentration range of 0.001 to 100 ng/mL. The linear part of the standard curve is shown in Figure 4b; the linear range of detection was 0.01–1.0 ng/mL with good correlation (R^2^ = 0.993) for ic-ELISA. From the standard calibration curve, the values of IC_50_ and LOD of 2-NP-AMOZ in ic-ELISA were calculated to be 0.11 and 0.009 ng/mL, respectively. Similarly, as shown in Figure 4b, the IC_50_ and LOD values for 2-NP-AMOZ by FLISA were 0.09 and 0.007 ng/mL, respectively, and the linear range was 0.01–1.0 ng/mL (R^2^ = 0.9918). The comparison between the two different detection approaches seemed to suggest that FLISA shows a higher detection sensitivity than ic-ELISA, most likely because of the more sensitive detection of fluorescence signals.

The specificity of the developed ic-ELISA was evaluated by cross-reactivity of the mAb with eleven structurally related 2-NP-AMOZ and the other two compounds. The cross-reactivities are summarized in Table 1. When the cross-reactivity with 2-NP-AMOZ was set to 100%, the cross-reactivities of mAb 5C10/3A8 with CPAMOZ, AMOZ, and Furaltadone were 122.2%, 10.7%, and 8.9%, respectively. There was no cross-reactivity (<0.01%) of the ic-ELISA with the other testing compounds.

### 3.4. Validation of the ic-ELISA and FLISA

The accuracy and precision of ic-ELISA and FLISA were determined using a variety of food matrices (including carp, salmon, shrimp, crab, swine muscle and liver, and chicken muscle and liver) spiked with AMOZ. As the mAb 5C10/3A8 detects only 2-NP-AMOZ but not AMOZ, the AMOZ residues were derivatized into 2-NP-AMOZ before detection using the immunoassay methods that we developed in this study. Different concentrations of AMOZ solutions at 0.5, 1.0, and 5.0 μg/kg were spiked in food samples, and the AMOZ was derivatized into 2-NP-AMOZ. The concentration of the 2-NP-AMOZ in the food matrix was determined and subsequently converted into AMOZ concentration according to the following formula: concentration of AMOZ = (molecular weight of AMOZ/molecular weight of 2-NP-AMOZ) × detected concentration of 2-NP-AMOZ. Table 2 shows the results of changes in the recoveries and coefficient of variation of the spiked experiments. For ic-ELISA analysis, the recoveries and coefficients of variation were 81.1–105.3% and 5.4–9.7%, respectively (n = 15). For FLISA analysis, the recoveries of 81.4–104.1% and coefficients of variation of 4.7–9.8% were obtained.

**Figure 5:**
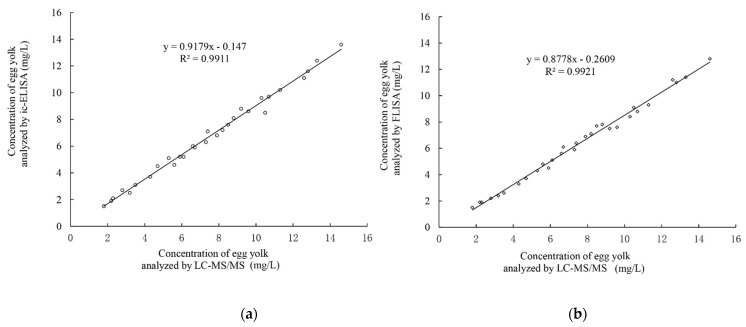
Correlations of the AMOZ assay between ic-ELISA and LC-MS/MS (**a**) and between FLISA and LC-MS/MS (**b**) for the incurred egg samples.

Thirty egg yolk samples from hens dosed with furaltadone were analyzed by the developed ic-ELISA and FLISA. Meanwhile, the concentrations of AMOZ residue in egg samples were detected following the reference LC-MS/MS. As shown in Figure 5, an excellent correlation between ic-ELISA and LC-MS/MS (R^2^ = 0.9911) and FLISA and LC-MS/MS (R^2^ = 0.9921) can be seen from the side-by-side comparison. These results suggest that the new ic-ELISA and FLISA measurements are reliable tools for the determination of AMOZ in real food samples.

### 3.5. Comparison of Published Detection Methods for AMOZ or 2-NP-AMOZ

Few reports exist about the immunoassay for AMOZ or 2-NP-AMOZ detection. We compared the ic-ELISA and FLISA methods with the only published paper that we found about immunoassays for the detection of AMOZ residue in animal tissues. The comparison results showed that Xu et al., Sheu et al., and Luo et. al. developed the polyclonal antibody (pAb)-based ic-ELISAs for the detection of 2-NP-AMOZ in tissue samples. However, the disadvantages of polyclonal antibodies are mainly its low sensitivity and high cross-reactivity, which limits its application [21,23,34]. It is well known that mAb can overcome these shortcomings. Based on different monoclonal antibodies, so many ic-ELISAs and fluoroimmunoassays have been developed to detect AMOZ in aquatic animals, beef, pork, and chicken. The IC_50_ values for 2-NP-AMOZ and AMOZ were 0.14–4.3 and 1.15–5.33 ng/mL in the homologous ic-ELISAs and fluoroimmunoassay, respectively. The IC_50_ and LODs of the proposed ic-ELISA and FLISA were slightly lower than the published ic-ELISAs, fluoroimmunoassay, and immunochromatographic assay-based colloidal gold method.

## 4. Conclusions

This study reported the development of mAb-based ic-ELISA and FLISA to evaluate the tissue-bound metabolite AMOZ by determining 2-NP-AMOZ. The IC_50_ values of ic-ELISA and FLISA for 2-NP-AMOZ were 0.11 and 0.09 ng/mL, respectively. For the two different detection modes, the recoveries ranged from 81.1% to 105.3% for spiked AMOZ blank tissue samples with coefficients of variation of less than 10%. The ic-ELISA and FLISA were also validated by LC-MS/MS. Relatively good correlations were obtained between the results of the proposed method and of the reference LC-MS/MS. These results indicate that the developed ic-ELISA and FLISA methods are feasible tools and can be used to detect AMOZ in edible animal tissues.

## Figures and Tables

**Table 1:** Cross-reactivity of mAb 5C10/3A8 with nitrofurans and other structurally related compounds by ic-ELISA.

Compounds	IC_50_ (ng/mL)	Cross-Reactivity (%)
2-NP-AMOZ	0.11	100
CPAMOZ	0.09	122.2
AMOZ	1.03	10.7
Furaltadone	1.24	8.9
Furazolidone	>1000	<0.01
AOZ	>1000	<0.01
2-NP-AOZ	>1000	<0.01
Nitrofurantoin	>1000	<0.01
AHD	>1000	<0.01
2-NP-AHD	>1000	<0.01
Nitrofurazone	>1000	<0.01
SEM	>1000	<0.01
2-NP-SEM	>1000	<0.01
2-NBA	>1000	<0.01
4-CBA	>1000	<0.01

**Table 2:** Mean recoveries and coefficient of variation of AMOZ in various biological matrices and the correlations (R^2^) between the results of ic-ELISA and FLISA.

Sample	Spiked (μg/kg)	Ic-ELISA		FLISA		The Correlations (R^2^)
		Recovery ± SD (%, n = 15)	CV (%, n = 15)	Recovery ± SD (%, n = 15)	CV (%, n = 15)	
Catfish	0.5	89.2 ± 8.4	9.5	92.6 ± 7.1	7.6	0.9652
	1.0	81.6 ± 6.3	7.7	85.7 ± 6.1	7.2	
	5.0	90.3 ± 8.0	8.8	92.1 ± 7.1	7.7	
Sturgeon	0.5	88.8 ± 7.4	8.4	91.8 ± 6.5	7.1	0.9758
	1.0	87.4 ± 8.3	9.5	90.0 ± 8.1	9.0	
	5.0	105.3 ± 10.2	9.7	103.6 ± 6.1	5.9	
Shrimp	0.5	87.8 ± 8.1	9.2	91.0 ± 7.8	8.6	0.9856
	1.0	81.1 ± 6.5	8.0	84.4 ± 6.5	7.7	
	5.0	82.4 ± 7.9	9.6	86.2 ± 6.5	7.6	
Crab	0.5	101.9± 7.6	7.5	104.1 ± 7.0	6.7	0.9927
	1.0	84.4 ± 6.1	7.2	87.4 ± 6.9	7.8	
	5.0	86.4 ±4.8	5.6	81.4 ± 5.9	7.2	
Swine muscle	0.5	88.9 ± 7.6	8.5	92.1 ± 7.5	8.1	0.9861
	1.0	95.4 ± 6.3	6.6	97.4 ± 6.0	6.1	
	5.0	85.9 ± 8.1	9.4	87.9 ± 8.7	9.8	
Swine liver	0.5	88.0 ± 5.5	6.3	91.1 ± 5.6	6.1	0.9835
	1.0	90.3 ± 7.9	8.8	93.1 ± 9.0	9.6	
	5.0	81.7 ± 5.9	7.2	83.9 ± 4.6	5.5	
Chicken muscle	0.5	91.9 ± 5.1	5.5	94.2 ± 4.6	4.9	0.9733
	1.0	83.2 ± 5.9	7.1	88.2 ± 6.0	6.9	
	5.0	83.6 ± 4.7	5.6	87.3 ± 4.1	4.7	
Chicken liver	0.5	85.1 ± 7.1	8.4	87.6 ± 7.4	8.5	0.9765
	1.0	89.8 ± 6.7	7.4	92.3 ± 7.1	7.7	
	5.0	87.9± 4.7	5.4	91.1 ± 4.3	4.8	

SD: standard deviation; CVs: coefficients of variation.

## Data Availability

The data presented in this study are available on request from the corresponding author.

## Sample Availability

Samples of the compounds are available from the authors.
